# Predictors of Outcome of Non–Muscle-Invasive and Muscle-Invasive Bladder Cancer

**DOI:** 10.1100/tsw.2011.28

**Published:** 2011-02-14

**Authors:** Ramy F. Youssef, Yair Lotan

**Affiliations:** Urology Department, UT Southwestern Medical Center, Dallas, Texas, USA

**Keywords:** bladder cancer, outcome, prognosis, bladder cancer markers

## Abstract

Bladder cancer is a major cause of morbidity and mortality. At initial diagnosis, 75% of patients present with non—muscle-invasive disease and 25% of patients have muscle-invasive or metastatic disease.Patients with noninvasive disease suffer from a high rate of recurrence and 10–30% will have disease progression. Patients with muscle-invasive disease are primarily treated with radical cystectomy, but frequently succumb to their disease despite improvements in surgical technique. In non–muscle-invasive disease, multiplicity, tumor size, and prior recurrence rates are the most important predictors for recurrence, while tumor grade, stage, and carcinoma *in situ* are the most important predictors for progression. The most common tool that clinicians use to predict outcomes after radical cystectomy is still the tumor-node-metastasis (TNM) staging system, with lymph node involvement representing the most important prognostic factor. However, the predictive accuracy of staging and grading systems are limited, and nomograms incorporating clinical and pathologic factors can improve prediction of bladder cancer outcomes. One limitation of current staging is the fact that tumors of a similar stage and grade can have significantly different biology. The integration of molecular markers, especially in a panel approach, has the potential to further improve the accuracy of predictive models and may also identify targets for therapeutic intervention or patients who will respond to systemic therapies.

### INTRODUCTION

Bladder cancer (BC) is the seventh most prevalent cancer worldwide, and results in significant morbidity and mortality. In the U.S., it is the fourth most common cancer in males and the ninth in females, with an estimated 70,980 new cases and 14,330 deaths in 2009[1]. At initial diagnosis, 75% present with non–muscle-invasive bladder cancer (NMIBC) and can be managed with transuretheral resection (TUR) and intravesical therapy. The remaining 25% present with muscle-invasive bladder cancer (MIBC) and the standard treatment is radical cystectomy (RC). The main problems of NMIBC are recurrence and progression, while MIBC is frequently associated with metastatic disease and is the major cause of mortality. Despite improvement in surgical techniques, 5-year disease-free survival (DFS) and cancer-specific survival (CSS) after RC remains between 50 and 70%[2,3,4]. Clinical understaging, micrometastasis, and underutilization of perioperative systemic therapies are among the reasons why RC alone might be insufficient to cure some patients[5,6].

Clinicians have used conventional clinicopathological parameters, such as tumor-node-metastasis (TNM) staging and grade of the tumor, as prognostic tools for patient counseling and treatment decisions. While these tools have provided useful estimates of survival outcome, the heterogeneity of tumor biology leads to large variation in outcomes within each stage and grade. Nomograms and prognostic modeling approaches simultaneously incorporating many factors have been developed for assessing the risk of individual patients[7,8,9,10,11]. Molecular markers, especially when incorporated in panels, can improve patient stratification and have the potential to enhance the accuracy of predictive models[12,13,14,15,16,17,18]. They can potentially identify targets for therapeutic intervention and patients who will respond to systemic therapies[19]. In this review, we highlight current predictors of outcome after management of both NMIBC and MIBC.

### PREDICTORS OF OUTCOME OF NON–MUSCLE-INVASIVE BLADDER CANCER

#### Clinicopathologic Factors Predicting Recurrence and Progression

In NMIBC, approximately 70% of patients present as pTa, 20% as pT1, and 10% with carcinoma *in situ* (CIS) lesions. Recurrence (50–80% of patients) is the main problem for pTa NMIBC patients, whereas progression (10–30% of patients) is the main threat in pT1 and CIS[20]. Multiplicity, tumor size, and prior recurrence rate are the most important predictors for recurrence[9,20]. Tumor grade, stage, and CIS may add in predicting recurrence, however, they are also the most important predictors for progression[9,20,21]. Sylvester et al. created a scoring system based on six variables to calculate the probability of recurrence (score from 0 to 17) and progression (score from 0 to 23) using data from 2,596 patients who participated in seven European Organization for Research and Treatment of Cancer (EORTC) trials. The number of tumors, tumor size, and prior recurrence rate obtained higher weights for calculating recurrence, while CIS, grade, and T category obtained higher weights for calculating progression[9]. The risk calculator is available at the EORTC Web site at www.eortc.be/tools/bladdercalculator. The European Association of Urology (EAU) defined patients at low, intermediate, and high risk for recurrence based on these scores and adopted this system in its guidelines[20,22]. The probabilities of recurrence at 1 and 5 years ranged from 15–61% and 31–78%, respectively. The probabilities of progression at 1 and 5 years ranged from <1–17% and 1–45%, respectively. The probability of recurrence at 1 year doubled from 15% for low-risk patients, to 24–38% for intermediate-risk patients, to 61% in high-risk patients. Moreover, the probability of progression at 5 years tripled from 6% in patients with low-intermediate risk, to 17% in high-intermediate risk, and to 45% in high-risk patients[9]. Multiplicity, prior tumor, CIS, and female gender were the significant predictors for recurrence in another large study that included 1,062 patients with NMIBC treated by bacillus Calmette-Guerin (BCG) collected from four randomized trials[21].

In summary; stage, grade, and the EORTC system can be useful when identifying high-risk patients. CIS, high-grade T1, and multiplicity represent high-risk factors. The advantage of the EORTC criteria is to help quantify risk, especially in intermediate-risk patients.

#### Treatment Factors

##### *Transurethral Resection*

The quality of TUR is an important factor when determining recurrence and progression of NMIBC[20,23,24]. A re-TUR is important in pT1 and high-grade NMIBC, and upstaging may occur in up to 30% of patients[20,24,25]. Moreover, patients with high-risk NMIBC may respond better to BCG

after re-TUR[26]. Grimm et al. found residual tumors in 27% of pTa and 53% of pT1 cases, of which 81% were at the initial resection site. Recurrence-free survival at the 5-year follow-up was 63% in the re-TUR group vs. 40% in the TUR-only group[27].

Further evidence for the importance of complete TUR comes from studies that demonstrated reduced recurrence rates in patients who underwent hexaminolevulinate-guided fluorescence cystoscopy compared to white light cystoscopy[28]. It is likely that a complete TUR at initial diagnosis or time of recurrence may reduce residual tumors. Furthermore, a re-TUR is critical in order to adequately stage patients with T1 high-grade disease since a significant proportion harbor residual invasive cancer.

### Intravesical Treatment

#### Perioperative Intravesical Therapy after TUR

Current guidelines recommend a single immediate postoperative chemotherapeutic instillation[22,29]. An immediate single instillation of a chemotherapeutic agent (within 24 h) after TUR decreased the incidence of recurrence in several randomized clinical trials where epirubicin and mitomycin C were the most commonly used drugs[30,31]. Perioperative intravesical therapy may treat missed tumors and reimplanted cells during TUR[32]. It may lead to 39% reduction in recurrence compared to TUR alone in patients with (Ta, T1) NMIBC[30]. However, the benefits of perioperative intravesical therapy may be more pronounced in low-risk patients and it was estimated that 8.5 patients need to be treated with peri-TUR chemotherapy to prevent one recurrence[20,30]. Thus, it should be considered just an initial therapy in intermediate- and high-risk patients. While most patients treated with perioperative chemotherapy experience mild side effects, it is contraindicated in cases where perforation is suspected.

#### Induction and Maintenance

Due to higher risks of recurrence and progression, intravesical therapy with BCG or chemotherapy is advocated in intermediate- and high-risk patients. BCG with maintenance has been shown to be superior to intravesical chemotherapy in several randomized trials[33,34,35,36,37]. Meta-analyses found a 32 and 27% reduction in risk of recurrence and progression on BCG[35,37]. Herr et al. reported that BCG increases the 10-year progression-free rate from 37 to 61.9% and DFS rate from 55 to 75%[38]. The optimal schedule and duration of intravesical therapy remain unknown. However, maintenance BCG seems to be beneficial, but its role in preventing progression in high-risk NMIBC is still controversial[20,34,35,36,39]. Despite the recommendations for use in the guidelines, there is still underutilization of intravesical therapy even in high-risk patients[29,40].

### Early Cystectomy vs. Conservative Management for High-Risk NMIBC

Randomized comparisons are lacking, but retrospective data show that BCG induction followed by maintenance is recommended in high-risk NMIBC after a second TUR[20]. RC is recommended in BCG failures (within a narrow time window) and is an option for patients with a higher risk for progression based on adverse prognostic factors: persistent pT1 disease in restaging second TUR; high-grade, concomitant CIS; micropapillary histology; solid architecture; and lymphovascular invasion[9,20,41,42,43,44,45]. A second course of BCG or intravesical chemotherapy may be an option in select patients or patients who refuse cystectomy[20]. Data from the Bladder Cancer Research Consortium (BCRC) showed that 50% of patients with clinical T1G3 disease were upstaged, 17% had nodal involvement at RC, and 19% died from BC after RC[46]. Nevertheless, TUR and adjuvant BCG

with the possibility of deferring RC seems a reasonable approach for the majority of high-risk patients as long as there is close follow-up and RC can be offered as soon as progression occurs[20].

### PREDICTORS OF OUTCOME OF MUSCLE-INVASIVE BLADDER CANCER

#### Pathological Tumor Characteristics

##### *Lymph Node Involvement*

The incidence of positive lymph nodes (LNs) in RC specimens is between 18 and 24%[2,3,4,47]. LN involvement may represent the most important prognostic feature after RC[48]. The presence of metastatic LNs is associated with poor outcome, with estimated 5-year survival rates of 20–35%[2,3,4,7]. LN involvement, the extent of lymphadenectomy (LND), the number of total LNs removed and counted by pathologist (even in node-negative patients), the number of positive nodes, and LN density were reported among the predictors of outcome after RC[48,49,50,51,52,53,54,55,56,57,58,59,60,61,62,63]. To date, there is still no exact definition for the standard LND during RC. The notion that extended LND minimizes the risk of leaving micrometastatic disease has been supported in several studies, which show that removing more LNs resulted in a better outcome[48,50,54,56,60,61,62,64]. Extended LND allows for more accurate staging and might add a therapeutic benefit. The ideal minimum number of LNs can only be determined in a randomized trial, but it has been suggested to be at least 15–20[48,49,51,57,63]. Recently, the Studer group used a multimodality technique and could accurately locate the primary lymphatic landing sites of the bladder. Limited pelvic LND removed only about 50% of all primary lymphatic landing sites, while extending the LND up to the ureteroiliac crossing removed 90%[64]. The template of LND might be more important than merely the number of LNs removed[61]. A very high level of extended LND might entail unnecessary risks that may benefit only very few patients[61,64].

##### *Tumor Stage*

Tumor stage is the second most important pathological predictor of outcome after RC[2,3,4]. Clinical staging prior to RC entails examination under anesthesia, pathological evaluation of TUR specimen, and preoperative radiographic imaging. In a study including 778 RC patients, pathological upstaging was found in 42% and downstaging in 22%[6]. The problem of understaging has significant implications on selection of patients for neoadjuvant chemotherapy. Definitive pathological evaluation after RC and LND may provide more accurate stratification of cancer-specific outcomes. Single-center reports as well as multi-institutional collaborations confirm the prognostic significance of primary pT stage. The 5-year DFS and CSS in patients with pT0-pT1, pT2, pT3, and pT4 is reported as 80–90, 50–80, 30–50, and 20–40%, respectively[2,3,4]. The importance of substaging of pT2 and pT3 has been recently validated[65,66].

Based on the high risk of recurrence and mortality in patients with T3 and T4 disease, a discussion regarding the benefits of adjuvant chemotherapy must be considered. Furthermore, surveillance protocols need to take final pathologic stage into consideration since patients with low-stage disease (T0-T1) may not need as intensive postoperative monitoring as those with more advanced disease.

##### *Grade*

While tumor grade is one of the most important predictors of recurrence and progression for NMIBC, it has less predictive power after RC[48]. The reason that grade is not significant in patients with muscle-invasive disease is that almost all patients have high-grade disease. However, Ghoneim et al. reported on a series of 2,720 consecutive RC cases and grade was the third independent prognostic factor after stage and nodal involvement[2]. Multiple grading systems have been published. The WHO/ISUP classification of 1998, which was updated in 2004, distinguishes low- and high-grade carcinomas, whereas the WHO 1973 system, which was updated in 1999, subdivides the high grade into grades II and III, and is otherwise very similar. The WHO/ISUP Consensus classification may provide a more simple and reproducible system for clinical practice[67,68].

#### *Lymphovascular Invasion*

Lymphovascular invasion (LVI) has been identified in 30–50% of RC specimens. It not only correlates with aggressive pathological features of BC, but also serves as a predictor of outcome independent of LN status[48,59,69,70,71,72,73,74,75]. This important distinction might improve the selection of patients for neoadjuvant and adjuvant chemotherapy adjunct to RC. Interestingly, LVI was found to be one of the prognostic factors for patients treated with RC for squamous cell carcinoma (SCC) of the bladder[76,77]. LVI might also serve as a valuable histological tool in the evaluation of TUR samples because there is significant agreement of LVI status at TUR and subsequent RC[73].

#### *Histological Subtypes*

In Western countries, transitional cell carcinoma (TCC) represents around 90% of BC, while SCC, adenocarcinoma, and other rare types represent the remaining 10%. However, SCC and adenocarcinoma are more common in areas where bilharziasis prevails, such as Egypt. Interestingly, while a multicenter study from the U.S. and Canada identified nontransitional/nonsquamous histology as an independent predictor of outcome after RC, Ghoneim et al., in their large study including 2,720 consecutive RC cases from Egypt, found no significant difference in outcome after RC between TCC, SCC, and adenocarcinoma cases[2,78]. The issue of different biological behaviors of different histological subtypes lies within our research focus. We have ongoing research efforts aimed at molecular characterization of different histological subtypes of BC[76,77,79,80,81]. Biomarkers may increase our understanding of the biological behavior of these cancers with potential use in treatment decisions and utilization of multimodal treatment approaches.

### Clinical Factors

#### *Times from Diagnosis to Surgery*

It was suggested that a delay in RC after diagnosis of MIBC for more than 3 months may be associated with worse outcomes[49,55,82,83]. However, there were some contradictory reports[84]. A delay in RC after diagnosis of MIBC may be justified if the patient is undergoing neoadjuvant therapy or needs to have optimization of medical condition prior to undergoing RC.

#### *Patient Age and Gender*

There is a growing body of evidence that RC remains the ideal therapy for MIBC and age alone should not preclude RC[55,85,86]. Chamie et al. used SEER data to compare radiotherapy and RC, and found significantly better median overall survival (OS) and CSS with RC for all groups, including the very elderly group (80–89 years of age)[87]. However, data from the BCRC showed that greater age was associated with an adverse outcome[88]. Women may have worse oncological outcomes after RC than men[55,89]. However, this cannot be confirmed from large cystectomy series[2,4].

### OUTCOME PREDICTION MODELS

#### Nomograms Based on Standard Histopathological Features

A nomogram is a graphical representation of a mathematical formula or algorithm that incorporates several prognostic factors to predict a particular end point. The International Bladder Cancer Consortium (IBCC) Nomogram included >9,000 patients from 12 centers to predict the risk of recurrence at 5 years after RC. Age, grade, pathological stage, histological type, LN status, and time from diagnosis to surgery were significant contributing factors in the nomogram. The predictive accuracy of the nomogram (75%) was significantly better than TNM staging (68%) or standard pathological grouping models (62%)[7]. The BCRC used a multi-institutional cohort of 731 consecutive RC patients to predict disease recurrence, cancer-specific mortality, and all-cause mortality at 2, 5, and 8 years after RC[8,10]. Pathological T and N stages, grade, LVI, CIS, and the utilization of neoadjuvant or adjuvant chemotherapy and/or radiation were significant contributing factors in the nomogram. The three developed nomograms exceeded the predictive accuracy of TNM staging with 78, 78, and 73% reported accuracy for the disease recurrence, cancer-specific mortality, and all-cause mortality nomograms, respectively. The BCRC and IBCC nomograms were externally validated in other cohorts[90].

Accurate preoperative prediction is essential for patient counseling, selection of patients for neoadjuvant chemotherapy, and appropriate design of clinical trials. Karakiewicz et al. developed a precystectomy nomogram for prediction of non–organ-confined disease (T3-T4 and/or N+) using patient age, TUR stage, grade, and the presence of CIS. The nomogram was more accurate than TUR stage in predicting advanced pathological stage and LN metastases[11].

#### *Incorporation of Biomarkers into Nomograms*

The heterogeneous biological behavior of tumors may limit the predictive accuracy of nomograms based on conventional clinicopathological features. A nomogram incorporating urinary NMP22, cytology, age, and gender could predict with high accuracy the probability of disease recurrence and progression in patients with NMIBC (available at www.nomogram.org)[91]. The integration of multiple biomarkers was found to improve BC prognostication[12,13,14,15,16,17]. Assessment of the number of altered biomarkers among a panel of p53, pRB, p21, p27, and cyclin E1 in RC specimens improved the prediction of recurrence and survival in patients with pTa-3N0M0 disease. Addition of the number of altered biomarkers significantly increased the predictive accuracy of nomograms based on the TNM staging system for disease recurrence and cancer-specific mortality by 10.9% (83.4 vs. 72.5%) and 8.6% (86.9 vs. 78.3%), respectively[14].

#### Artificial Neural Networks

Artificial neural networks (ANN) are algorithms that can be trained to recognize complex patterns in datasets. They have an advantage over conventional statistics in that they are not constrained by predefined mathematical relationships between dependent and independent variables; thus, they are able to model complex nonlinear parameters[92]. Recently, el-Mekresh et al. published that ANN outperformed risk group stratification model and nomogram construction in predicting 5-year survival probabilities after RC[93]. Two other studies added biomarkers to standard clinicopathological features, using ANN and neurofuzzy modeling[92,94]. However, to date, the use of these models is still far from clinical practice.

## MOLECULAR PREDICTORS OF OUTCOME OF NONINVASIVE AND INVASIVE BLADDER CANCER

### Cell Cycle Regulators

The most intensively studied molecular biomarker for BC is p53. It plays a vital role in the regulation of cell cycle and is important for genetic stability, cell proliferation, apoptosis, and inhibition of angiogenesis[95]. A defect in p53 leads to loss of p53-dependent apoptosis and gives a proliferation advantage. Many studies have suggested that p53 can predict poor outcome of both NMIBC and MIBC. In 1994, Esrig et al. published that p53 at the time of RC in patients with organ-confined node-negative disease independently predicts a significantly increased risk of recurrence and death[96]. However, there are some contradictory reports regarding the prognostic value of p53 in BC[17,97,98]. A large multicenter adjuvant chemotherapy study that randomized patients to adjuvant chemotherapy vs. observation based on p53 status could not so far confirm the prognostic value of p53[28]. Other studies found that altered p53 gradually increased from normal urothelium to NMIBC to CIS to MIBC, and was highest in metastatic LNs[12,15,16,99]. Moreover, p53 expression was significantly associated with tumor stage, grade, LVI, LN metastases, disease recurrence, and BC-specific death. Interestingly, p53 was the strongest predictor of BC outcomes in patients treated with RC among a panel including p21, pRB, p27, p16, and cyclin E1[12,15,16,99]. We recently highlighted the prognostic value of p53 in SCC of the bladder as well. P53 was the only prognostic marker in patients treated with RC for SCC among a panel including p53, p21, p27, cyclin E, and Ki-67[77].

Wild-type p53 protein induces p21 protein, a product of the waf1/cip1 gene. P21 and p27 are downstream cyclin-dependent kinase inhibitors that inhibit cells from entering the G1 to S phase[17]. P53, p21, and p27 have been implicated in the oncogenesis, progression, and outcome of BC[13,14,16,100,101,102,103]. P21 status is an independent predictor of recurrence and survival after RC[12,15]. Moreover, patients with p53-altered/p21-negative tumors have a higher rate of recurrence and worse survival than those with p53-altered/p21-positive tumors[104]. P27 was the second most powerful cell cycle regulator after p53 for prediction of recurrence and survival in patients with MIBC treated with RC[12,15]. However, it has limited predictive value in patients with NMIBC[16,17]. Furthermore, the status of individual cell cycle regulators did not add significantly to predictions of outcome in patients with very advanced disease (node positive and T4)[105].

Ki-67 expression is a measure of the cell growth and, hence, biological aggressiveness of a cancer. In MIBC, Ki-67 overexpression was significantly associated with advanced pathological stage, higher tumor grade, LVI, and metastases to LNs, as well as both disease recurrence and BC-specific mortality[12,48,106,107]. We recently found a prognostic role for combined p27 and Ki-67 alterations in adenocarcinoma of the bladder[81].

Cyclin E deregulations are common events in cancer, regardless of the tumor origin. Cyclin E alterations can enhance tumor progression through speeding of the $G_1$ phase. Cyclin E was shown to be associated with advanced pathologic stage, LVI, LN metastases, and BC-specific mortality after RC[99]. On the other hand, cyclin D was not a predictor of outcome in patients with BC[99].

Inactivation of pRB is a major mechanism by which cancer cells attain a growth advantage during tumorigenesis. However, the predictive power of pRB may be inferior to other cell cycle regulators both in NMIBC and MIBC[17].

### Apoptotic Markers

Caspase-3 was found to be associated with higher pathologic grade, stage, LN metastasis, disease recurrence, and CSS after RC[17,18,48]. Bcl-2 is another apoptotic marker that controls ion channels, caspase status, and cytochrome c location. Bcl-2, caspase-3, p53, and survivin have a cooperative effect on progression of BC. Assessment of these combined apoptosis markers status and number of altered markers in patients treated by RC provides prognostic information that could help in the prediction of disease recurrence and mortality[18].

Survivin overexpression is also an important apoptotic marker associated with higher pathologic stage, LVI, LN metastasis, recurrence, and CSS in patients treated with RC[17,18,108]. In addition, Karam et al. found that survivin expression analysis performed on TUR specimens might identify patients with NMIBC at high risk of disease recurrence and progression who would benefit from closer follow-up or more aggressive therapy[109]. Moreover, survivin overexpression increased gradually from NMIBC to advanced BC to metastatic LN tissue[109,110].

### Angiogenesis Markers

The vascular endothelial growth factor (VEGF) family is an important determinant of angiogenesis. VEGF promotes endothelial mitogenesis and migration, extracellular matrix remodeling, increased vascular permeability, and maintenance of newly formed vasculature. Higher VEGF expression was associated with increasing tumor stage, grade, progression, and recurrence in patients treated with TUR[17,19]. VEGF expression is not only associated with tumor size, stage, and grade, but also with LVI, LN metastasis, and worse overall prognosis[111]. Increased VEGF levels can result in increased vascular permeability and interstitial fluid pressure, impairing chemotherapy delivery. Adding anti-VEGF to chemotherapeutic regimens might lead to improved responses[112]. Recently, we showed that VEGF was overexpressed in a large number of patients treated with RC for urothelial carcinoma of the bladder (86%). It was not only associated with pathological features, but also with altered expression of p21, p27, RB, cyclin E1, and Ki-67, suggesting complex interactions between different pathways. These findings support the role of VEGF in bladder tumorigenesis and further support it as a potential target for therapy[105].

Thrombospondin-1 (TSP-1) is a potent inhibitor of angiogenesis that is independently associated with disease recurrence and all-cause mortality after RC[17,19,105,113]. Recently, we investigated the utility of angiogenesis-related molecular markers in the prediction of the clinical outcome after RC. TSP-1 was the only independent predictor of disease recurrence as well as cancer-specific mortality[105]. Grossfeld et al. previously reported that tumors with p53 alterations are associated with low TSP-1 expression, and these tumors are more likely to demonstrate high microvessel density (MVD) counts[113]. MVD, a surrogate marker for angiogenesis, has also been demonstrated to be a prognostic marker associated with highest risk of recurrence and BC-specific mortality in MIBC[19,113,114]. In our recent study of angiogenesis markers, MVD was the only molecular marker associated with p53 alterations. We also showed that MVD was higher in patients with LN metastasis, which was proven in earlier studies as well[105,115].

### Combination of Molecular Markers

Assessment of any single molecular biomarker may not adequately reflect tumor biology or provide reliable prognostic stratification due to the multistep tumorigenesis and complexity of molecular alterations in BC. There is a strong trend towards simultaneous assessment of multiple biomarkers and panels were found to be superior to single-marker assessment, in terms of prognostic value, in both MIBC as well as NMIBC[12,13,14,15,16,17,18]. Since prospective validation is an important step prior to widespread clinical utilization of markers, we have initiated, since January 2007, a prospective validation of a marker panel in predicting outcomes after RC and upstaging at time of RC. The preliminary analysis of our ongoing prospective trial strongly suggests that a panel of five biomarkers not only predicts poor outcome after RC, but also improves the identification of patients at risk of upstaging at RC. An unfavorable prognostic score may identify patients who are most likely to benefit from neoadjuvant and adjuvant chemotherapy combined with RC[116].

### CONCLUSIONS

Bladder cancer is a heterogeneous disease that is insufficiently characterized by conventional clinicopathological factors. Precise prediction of outcome is essential for counseling patients, selecting them for neoadjuvant and adjuvant systemic therapies, and determining their eligibility for clinical trials. The TNM staging system has been validated and used universally to predict outcome after RC. However, it has limited ability to predict tumor recurrence or patient survival. Nomograms are a highly appealing means that have provided better individualized risk estimates to facilitate treatment decisions. Biomarkers may help to elucidate unique biologic features to identify patients at high risk for progression after local treatment, upstaging at time of RC, or poor outcome after RC. Prospective trials based on alterations of markers panel must be designed to validate the promising data on molecular biomarkers for BC.

## References

1. Jemal, A., Siegel, R., Ward, E., et al. (2009) Cancer statistics, 2009. *CA Cancer J. Clin.* **59,** 225.
2. Ghoneim, M.A., Abdel-Latif, M., el-Mekresh, M., et al. (2008) Radical cystectomy for carcinoma of the bladder: 2,720 consecutive cases 5 years later. *J. Urol.* **180,** 121.
3. Shariat, S.F., Karakiewicz, P.I., Palapattu, G.S., et al. (2006) Outcomes of radical cystectomy for transitional cell carcinoma of the bladder: a contemporary series from the Bladder Cancer Research Consortium. *J. Urol.* **176,** 2414.
4. Stein, J.P., Lieskovsky, G., Cote, R., et al. (2001) Radical cystectomy in the treatment of invasive bladder cancer: long-term results in 1,054 patients. *J. Clin. Oncol.* **19,** 666.
5. David, K.A., Milowsky, M.I., Ritchey, J., et al. (2007) Low incidence of perioperative chemotherapy for stage III bladder cancer 1998 to 2003: a report from the National Cancer Data Base. *J. Urol.* **178,** 451.
6. Shariat, S.F., Palapattu, G.S., Karakiewicz, P.I., et al. (2007) Discrepancy between clinical and pathologic stage: impact on prognosis after radical cystectomy. *Eur. Urol.* **51,** 137.
7. Bochner, B.H., Kattan, M.W., and Vora, K.C. (2006) Postoperative nomogram predicting risk of recurrence after radical cystectomy for bladder cancer. *J. Clin. Oncol.* **24,** 3967.
8. Shariat, S.F., Karakiewicz, P.I., Palapattu, G.S., et al. (2006) Nomograms provide improved accuracy for predicting survival after radical cystectomy. *Clin. Cancer Res.* **12,** 6663.
9. Sylvester, R.J., van der Meijden, A.P., Oosterlinck, W., et al. (2006) Predicting recurrence and progression in individual patients with stage Ta T1 bladder cancer using EORTC risk tables: a combined analysis of 2596 patients from seven EORTC trials. *Eur. Urol.* **49,** 466.
10. Karakiewicz, P.I., Shariat, S.F., Palapattu, G.S., et al. (2006) Nomogram for predicting disease recurrence after radical cystectomy for transitional cell carcinoma of the bladder. *J. Urol.* **176,** 1354.
11. Karakiewicz, P.I., Shariat, S.F., Palapattu, G.S., et al. (2006) Precystectomy nomogram for prediction of advanced bladder cancer stage. *Eur. Urol.* **50,** 1254.
12. Shariat, S.F., Bolenz, C., Godoy, G., et al. (2009) Predictive value of combined immunohistochemical markers in patients with pT1 urothelial carcinoma at radical cystectomy. *J. Urol.* **182,** 78.
13. Shariat, S.F., Chade, D.C., Karakiewicz, P.I., et al. (2010) Combination of multiple molecular markers can improve prognostication in patients with locally advanced and lymph node positive bladder cancer. *J. Urol.* **183,** 68.
14. Shariat, S.F., Karakiewicz, P.I., Ashfaq, R., et al. (2008) Multiple biomarkers improve prediction of bladder cancer recurrence and mortality in patients undergoing cystectomy. *Cancer* **112,** 315.
15. Shariat, S.F., Zlotta, A.R., Ashfaq, R., et al. (2007) Cooperative effect of cell-cycle regulators expression on bladder cancer development and biologic aggressiveness. *Mod. Pathol.* **20,** 445.
16. Shariat, S.F., Ashfaq, R., Sagalowsky, A.I., et al. (2007) Predictive value of cell cycle biomarkers in nonmuscle invasive bladder transitional cell carcinoma. *J. Urol.* **177,** 481.
17. Shariat, S.F., Karam, J.A., and Lerner, S.P. (2008) Molecular markers in bladder cancer. *Curr. Opin. Urol.* **18,** 1.
18. Karam, J.A., Lotan, Y., Karakiewicz, P.I., et al. (2007) Use of combined apoptosis biomarkers for prediction of bladder cancer recurrence and mortality after radical cystectomy. *Lancet Oncol.* **8,** 128.
19. Youssef, R.F., Mitra, A.P., Bartsch, G., Jr., et al. (2009) Molecular targets and targeted therapies in bladder cancer management. *World J. Urol.* **27,** 9.
20. van Rhijn, B.W., Burger, M., Lotan, Y., et al. (2009) Recurrence and progression of disease in non-muscle-invasive bladder cancer: from epidemiology to treatment strategy. *Eur. Urol.* **56,** 430.
21. Fernandez-Gomez, J., Solsona, E., Unda, M., et al. (2008) Prognostic factors in patients with non-muscle-invasive bladder cancer treated with bacillus Calmette-Guerin: multivariate analysis of data from four randomized CUETO trials. *Eur. Urol.* **53,** 992.
22. Babjuk, M., Oosterlinck, W., Sylvester, R., et al. (2008) EAU guidelines on non-muscle-invasive urothelial carcinoma of the bladder. *Eur. Urol.* **54,** 303.

23. Brausi, M., Collette, L., Kurth, K., et al. (2002) Variability in the recurrence rate at first follow-up cystoscopy after TUR in stage Ta T1 transitional cell carcinoma of the bladder: a combined analysis of seven EORTC studies. *Eur. Urol.* **41,** 523.
24. Herr, H.W. and Donat, S.M. (2008) Quality control in transurethral resection of bladder tumours. *BJU Int.* **102,** 1242.
25. Jakse, G., Algaba, F., Malmstrom, P.U., et al. (2004) A second-look TUR in T1 transitional cell carcinoma: why? *Eur. Urol.* **45,** 539.
26. Herr, H.W. (2005) Restaging transurethral resection of high risk superficial bladder cancer improves the initial response to bacillus Calmette-Guerin therapy. *J. Urol.* **174,** 2134.
27. Grimm, M.O., Steinhoff, C., Simon, X., et al. (2003) Effect of routine repeat transurethral resection for superficial bladder cancer: a long-term observational study. *J. Urol.* **170,** 433.
28. https://www.uscnorris.com/p53/
29. Hall, M.C., Chang, S.S., Dalbagni, G., et al. (2007) Guideline for the management of nonmuscle invasive bladder cancer (stages Ta, T1, and Tis): 2007 update. *J. Urol.* **178,** 2314.
30. Sylvester, R.J., Oosterlinck, W., and van der Meijden, A.P. (2004) A single immediate postoperative instillation of chemotherapy decreases the risk of recurrence in patients with stage Ta T1 bladder cancer: a meta-analysis of published results of randomized clinical trials. *J. Urol.* **171,** 2186.
31. Gudjonsson, S., Adell, L., Merdasa, F., et al. (2009) Should all patients with non-muscle-invasive bladder cancer receive early intravesical chemotherapy after transurethral resection? The results of a prospective randomised multicentre study. *Eur. Urol.* **55,** 773.
32. Kamat, A.M. and Lotan, Y.R. (2010) Perioperative intravesical therapy after transurethral resection for bladder cancer. *J. Urol.* **183,** 19.
33. Bohle, A., Jocham, D., and Bock, P.R. (2003) Intravesical bacillus Calmette-Guerin versus mitomycin C for superficial bladder cancer: a formal meta-analysis of comparative studies on recurrence and toxicity. *J. Urol.* **169,** 90.
34. Gontero, P., Bohle, A., Malmstrom, P.U., et al. (2010) The role of bacillus Calmette-Guerin in the treatment of non-muscle-invasive bladder cancer. *Eur. Urol.* **57,** 410.
35. Malmstrom, P.U., Sylvester, R.J., Crawford, D.E., et al. (2009) An individual patient data meta-analysis of the long-term outcome of randomised studies comparing intravesical mitomycin C versus bacillus Calmette-Guerin for non-muscle-invasive bladder cancer. *Eur. Urol.* **56,** 247.
36. Sylvester, R.J. (2009) Bacillus Calmette-Guerin versus mitomycin C for the treatment of intermediate-risk non-muscle-invasive bladder cancer: the debate continues. *Eur. Urol.* **56,** 266.
37. Sylvester, R.J., van der, M.A., and Lamm, D.L. (2002) Intravesical bacillus Calmette-Guerin reduces the risk of progression in patients with superficial bladder cancer: a meta-analysis of the published results of randomized clinical trials. *J. Urol.* **168,** 1964.
38. Herr, H.W., Schwalb, D.M., Zhang, Z.F., et al. (1995) Intravesical bacillus Calmette-Guerin therapy prevents tumor progression and death from superficial bladder cancer: ten-year follow-up of a prospective randomized trial. *J. Clin. Oncol.* **13,** 1404.
39. Herr, H.W. (2008) Is maintenance Bacillus Calmette-Guerin really necessary? *Eur. Urol.* **54,** 971.
40. Huang, G.J., Hamilton, A.S., Lo, M., et al. (2008) Predictors of intravesical therapy for nonmuscle invasive bladder cancer: results from the surveillance, epidemiology and end results program 2003 patterns of care project. *J. Urol.* **180,** 520.
41. Masood, S., Sriprasad, S., Palmer, J.H., et al. (2004) T1G3 bladder cancer--indications for early cystectomy. *Int. Urol. Nephrol.* **36,** 41.
42. Kamat, A.M., Dinney, C.P., Gee, J.R., et al. (2007) Micropapillary bladder cancer: a review of the University of Texas M. D. Anderson Cancer Center experience with 100 consecutive patients. *Cancer* **110,** 62.
43. Denzinger, S., Otto, W., Fritsche, H.M., et al. (2007) Bladder sparing approach for initial T1G3 bladder cancer: do multifocality, size of tumor or concomitant carcinoma in situ matter? A long-term analysis of 132 patients. *Int. J. Urol.* **14,** 995.
44. Andius, P., Johansson, S.L., and Holmang, S. (2007) Prognostic factors in stage T1 bladder cancer: tumor pattern (solid or papillary) and vascular invasion more important than depth of invasion. *Urology* **70,** 758.
45. Herr, H.W., Donat, S.M., and Dalbagni, G. (2007) Can restaging transurethral resection of T1 bladder cancer select patients for immediate cystectomy? *J. Urol.* **177,** 75.
46. Gupta, A., Lotan, Y., Bastian, P.J., et al. (2008) Outcomes of patients with clinical T1 grade 3 urothelial cell bladder carcinoma treated with radical cystectomy. *Urology* **71,** 302.
47. Hautmann, R.E., Gschwend, J.E., de Petriconi, R.C., et al. (2006) Cystectomy for transitional cell carcinoma of the bladder: results of a surgery only series in the neobladder era. *J. Urol.* **176,** 486.
48. Margulis, V., Lotan, Y., Montorsi, F., et al. (2008) Predicting survival after radical cystectomy for bladder cancer. *BJU Int.* **102,** 15.
49. Ghoneim, M.A. and Abol-Enein, H. (2008) Management of muscle-invasive bladder cancer: an update. *Nat. Clin. Pract. Urol.* **5,** 501.
50. Leissner, J., Ghoneim, M.A., Abol-Enein, H., et al. (2004) Extended radical lymphadenectomy in patients with urothelial bladder cancer: results of a prospective multicenter study. *J. Urol.* **171,** 139.

51. Ghoneim, M.A. and Abol-Enein, H. (2004) Lymphadenectomy with cystectomy: is it necessary and what is its extent? *Eur. Urol.* **46,** 457.
52. Abol-Enein, H., El-Baz, M., Abd El-Hameed, M.A., et al. (2004) Lymph node involvement in patients with bladder cancer treated with radical cystectomy: a patho-anatomical study--a single center experience. *J. Urol.* **172,** 1818.
53. Abdel-Latif, M., Abol-Enein, H., El-Baz, M., et al. (2004) Nodal involvement in bladder cancer cases treated with radical cystectomy: incidence and prognosis. *J. Urol.* **172,** 85.
54. Herr, H.W., Bochner, B.H., Dalbagni, G., et al. (2002) Impact of the number of lymph nodes retrieved on outcome in patients with muscle invasive bladder cancer. *J. Urol.* **167,** 1295.
55. Thalmann, G.N. and Stein, J.P. (2008) Outcomes of radical cystectomy. *BJU Int.* **102,** 1279.
56. Stein, J.P. and Skinner, D.G. (2005) The role of lymphadenectomy in high-grade invasive bladder cancer. *Urol. Clin. North Am.* **32,** 187.
57. Stein, J.P. (2007) The role of lymphadenectomy in patients undergoing radical cystectomy for bladder cancer. *Curr. Oncol. Rep.* **9,** 213.
58. Stein, J.P. (2006) Lymphadenectomy in bladder cancer: how high is "high enough"? *Urol. Oncol.* **24,** 349.
59. Quek, M.L., Stein, J.P., Nichols, P.W., et al. (2005) Prognostic significance of lymphovascular invasion of bladder cancer treated with radical cystectomy. *J. Urol.* **174,** 103.
60. Lerner, S.P. (2009) The role and extent of pelvic lymphadenectomy in the management of patients with invasive urothelial carcinoma. *Curr. Treat. Options Oncol.* **10,** 267.
61. Herr, H.W. (2010) Extent of pelvic lymph node dissection during radical cystectomy: where and why! *Eur. Urol.* **57,** 212.
62. Herr, H.W. (2007) The concept of lymph node density--is it ready for clinical practice? *J. Urol.* **177,** 1273.
63. Stein, J.P., Cai, J., Groshen, S., et al. (2003) Risk factors for patients with pelvic lymph node metastases following radical cystectomy with en bloc pelvic lymphadenectomy: concept of lymph node density. *J. Urol.* **170,** 35.
64. Roth, B., Wissmeyer, M.P., Zehnder, P., et al. (2010) A new multimodality technique accurately maps the primary lymphatic landing sites of the bladder. *Eur. Urol.* **57,** 205.
65. Tilki, D., Svatek, R.S., Karakiewicz, P.I., et al. (2010) pT3 substaging is a prognostic indicator for lymph node negative urothelial carcinoma of the bladder. *J. Urol.* **184,** 470.
66. Tilki, D., Reich, O., Karakiewicz, P.I., et al. (2010) Validation of the AJCC TNM substaging of pT2 bladder cancer: deep muscle invasion is associated with significantly worse outcome. *Eur. Urol.* **58,** 112.
67. Busch, C. and Algaba, F. (2002) The WHO/ISUP 1998 and WHO 1999 systems for malignancy grading of bladder cancer. Scientific foundation and translation to one another and previous systems. *Virchows Arch*. **441,** 105.
68. Miyamoto, H., Miller, J.S., Fajardo, D.A., et al. (2010) Non-invasive papillary urothelial neoplasms: the 2004 WHO/ISUP classification system. *Pathol. Int.* **60,** 1.
69. Lotan, Y., Gupta, A., Shariat, S.F., et al. (2005) Lymphovascular invasion is independently associated with overall survival, cause-specific survival, and local and distant recurrence in patients with negative lymph nodes at radical cystectomy. *J. Clin. Oncol.* **23,** 6533.
70. Kikuchi, E., Margulis, V., Karakiewicz, P.I., et al. (2009) Lymphovascular invasion predicts clinical outcomes in patients with node-negative upper tract urothelial carcinoma. *J. Clin. Oncol.* **27,** 612.
71. Bolenz, C., Herrmann, E., Bastian, P.J., et al. (2010) Lymphovascular invasion is an independent predictor of oncological outcomes in patients with lymph node-negative urothelial bladder cancer treated by radical cystectomy: a multicentre validation trial. *BJU Int.* **106,** 493.
72. Canter, D., Guzzo, T., Resnick, M., et al. (2008) The presence of lymphovascular invasion in radical cystectomy specimens from patients with urothelial carcinoma portends a poor clinical prognosis. *BJU Int.* **102,** 952.
73. Kunju, L.P., You, L., Zhang, Y., et al. (2008) Lymphovascular invasion of urothelial cancer in matched transurethral bladder tumor resection and radical cystectomy specimens. *J. Urol.* **180,** 1928.
74. Manoharan, M., Katkoori, D., Kishore, T.A., et al. (2010) Lymphovascular invasion in radical cystectomy specimen: is it an independent prognostic factor in patients without lymph node metastases? *World J. Urol.* **28,** 233.
75. Shariat, S.F., Svatek, R.S., Tilki, D., et al. (2010) International validation of the prognostic value of lymphovascular invasion in patients treated with radical cystectomy. *BJU Int.* **105,** 1402.
76. Youssef, R.F., Kapur, P., Kabbani, W., et al. (2010) Expression of cyclooxygenase-2 in squamous cell carcinoma of the urinary bladder. AUA Meeting 2010 Abstracts. *J. Urol. Suppl.* **183,** e447.
77. Youssef, R.F., Shariat, S.F., Kapur, P., et al. (2010) Expression of cell cycle-related molecular markers in patients treated with radical cystectomy for squamous cell carcinoma of the bladder. *Hum. Pathol.* [Epub ahead of print]
78. Rogers, C.G., Palapattu, G.S., Shariat, S.F., et al. (2006) Clinical outcomes following radical cystectomy for primary nontransitional cell carcinoma of the bladder compared to transitional cell carcinoma of the bladder. *J. Urol.* **175,** 2048.
79. Youssef, R.F., Kapur, P., Kabbani, W., et al. (2010) Bilharzial related bladder cancer: pathological characteristics and value of cyclooxygenase-2 expression. AUA Meeting 2010 Abstracts. *J. Urol. Suppl.* **183,** e663.
80. Youssef, R.F., Kapur, P., Kabbani, W., et al. (2010) Bilharzial vs non-bilharzial bladder cancer: pathological characteristics and value of cyclooxgenase-2 expression. *BJU Int.* [Epub ahead of print]
81. King, E., Youssef, R.F., Kabbani, W., et al. (2010) Adenocarcinoma of the urinary bladder: value of cell cycle biomarkers. In United States and Canadian Academy of Pathology, 99th Annual Meeting, Washington, D.C.

82. Chang, S.S., Hassan, J.M., Cookson, M.S., et al. (2003) Delaying radical cystectomy for muscle invasive bladder cancer results in worse pathological stage. *J. Urol.* **170,** 1085.
83. Sanchez-Ortiz, R.F., Huang, W.C., Mick, R., et al. (2003) An interval longer than 12 weeks between the diagnosis of muscle invasion and cystectomy is associated with worse outcome in bladder carcinoma. *J. Urol.* **169,** 110.
84. Nielsen, M.E., Palapattu, G.S., Karakiewicz, P.I., et al. (2007) A delay in radical cystectomy of >3 months is not associated with a worse clinical outcome. *BJU Int.* **100,** 1015.
85. Froehner, M., Brausi, M.A., Herr, H.W., et al. (2009) Complications following radical cystectomy for bladder cancer in the elderly. *Eur. Urol.* **56,** 443.
86. Clark, P.E., Stein, J.P., Groshen, S.G., et al. (2005) Radical cystectomy in the elderly: comparison of clincal outcomes between younger and older patients. *Cancer* **104,** 36.
87. Chamie, K., Hu, B., Devere White, R.W., et al. (2008) Cystectomy in the elderly: does the survival benefit in younger patients translate to the octogenarians? *BJU Int.* **102,** 284.
88. Nielsen, M.E., Shariat, S.F., Karakiewicz, P.I., et al. (2007) Advanced age is associated with poorer bladder cancer-specific survival in patients treated with radical cystectomy. *Eur. Urol.* **51,** 699.
89. Tilki, D., Svatek, R.S., Karakiewicz, P.I., et al. (2010) Characteristics and outcomes of patients with pT4 urothelial carcinoma at radical cystectomy: a retrospective international study of 583 patients. *J. Urol.* **183,** 87.
90. Zaak, D., Burger, M., Otto, W., et al. (2009) Predicting individual outcomes after radical cystectomy: an external validation of current nomograms. *BJU Int.* **106,** 342.
91. Shariat, S.F., Zippe, C., Ludecke, G., et al. (2005) Nomograms including nuclear matrix protein 22 for prediction of disease recurrence and progression in patients with Ta, T1 or CIS transitional cell carcinoma of the bladder. *J. Urol.* **173,** 1518.
92. Qureshi, K.N., Naguib, R.N., Hamdy, F.C., et al. (2000) Neural network analysis of clinicopathological and molecular markers in bladder cancer. *J. Urol.* **163,** 630.
93. el-Mekresh, M., Akl, A., Mosbah, A., et al. (2009) Prediction of survival after radical cystectomy for invasive bladder carcinoma: risk group stratification, nomograms or artificial neural networks? *J. Urol.* **182,** 466.
94. Catto, J.W., Linkens, D.A., Abbod, M.F., et al. (2003) Artificial intelligence in predicting bladder cancer outcome: a comparison of neuro-fuzzy modeling and artificial neural networks. *Clin. Cancer Res*. **9,** 4172.
95. Vogelstein, B., Lane, D., and Levine, A.J. (2000) Surfing the p53 network. *Nature* **408,** 307.
96. Esrig, D., Elmajian, D., Groshen, S., et al. (1994) Accumulation of nuclear p53 and tumor progression in bladder cancer. *N. Engl. J. Med.* **331,** 1259.
97. Malats, N., Bustos, A., Nascimento, C.M., et al. (2005) P53 as a prognostic marker for bladder cancer: a meta-analysis and review. *Lancet Oncol.* **6,** 678.
98. Bolenz, C. and Lotan, Y. (2008) Molecular biomarkers for urothelial carcinoma of the bladder: challenges in clinical use. *Nat. Clin. Pract. Urol.* **5,** 676.
99. Shariat, S.F., Ashfaq, R., Sagalowsky, A.I., et al. (2006) Correlation of cyclin D1 and E1 expression with bladder cancer presence, invasion, progression, and metastasis. *Hum. Pathol.* **37,** 1568.
100. Shariat, S.F., Lotan, Y., Karakiewicz, P.I., et al. (2009) p53 predictive value for pT1-2 N0 disease at radical cystectomy. *J. Urol.* **182,** 907.
101. Shariat, S.F., Bolenz, C., Karakiewicz, P.I., et al. (2010) p53 expression in patients with advanced urothelial cancer of the urinary bladder. *BJU Int.* **105,** 489.
102. Shariat, S.F., Tokunaga, H., Zhou, J., et al. (2004) p53, p21, pRB, and p16 expression predict clinical outcome in cystectomy with bladder cancer. *J. Clin. Oncol.* **22,** 1014.
103. Shariat, S.F., Kim, J., Raptidis, G., et al. (2003) Association of p53 and p21 expression with clinical outcome in patients with carcinoma in situ of the urinary bladder. *Urology* **61,** 1140.
104. Stein, J.P., Ginsberg, D.A., Grossfeld, G.D., et al. (1998) Effect of p21WAF1/CIP1 expression on tumor progression in bladder cancer. *J. Natl. Cancer Inst*. **90,** 1072.
105. Shariat, S.F., Youssef, R.F., Gupta, A., et al. (2010) Association of angiogenesis related markers with bladder cancer outcomes and other molecular markers. *J. Urol.* **183,** 1744.
106. Margulis, V., Lotan, Y., Karakiewicz, P.I., et al. (2009) Multi-institutional validation of the predictive value of Ki-67 labeling index in patients with urinary bladder cancer. *J. Natl. Cancer Inst.* **101,** 114.
107. Margulis, V., Shariat, S.F., Ashfaq, R., et al. (2006) Ki-67 is an independent predictor of bladder cancer outcome in patients treated with radical cystectomy for organ-confined disease. *Clin. Cancer Res.* **12,** 7369.
108. Shariat, S.F., Karakiewicz, P.I., Godoy, G., et al. (2009) Survivin as a prognostic marker for urothelial carcinoma of the bladder: a multicenter external validation study. *Clin. Cancer Res.* **15,** 7012.
109. Karam, J.A., Lotan, Y., Ashfaq, R., et al. (2007) Survivin expression in patients with non-muscle-invasive urothelial cell carcinoma of the bladder. *Urology* **70,** 482.
110. Shariat, S.F., Ashfaq, R., Karakiewicz, P.I., et al. (2007) Survivin expression is associated with bladder cancer presence, stage, progression, and mortality. *Cancer* **109,** 1106.
111. Zu, X., Tang, Z., Li, Y., et al. (2006) Vascular endothelial growth factor-C expression in bladder transitional cell cancer and its relationship to lymph node metastasis. *BJU Int.* **98,** 1090.
112. Elfiky, A.A. and Rosenberg, J.E. (2009) Targeting angiogenesis in bladder cancer. *Curr. Oncol. Rep.* **11,** 244.

113. Grossfeld, G.D., Ginsberg, D.A., Stein, J.P., et al. (1997) Thrombospondin-1 expression in bladder cancer: association with p53 alterations, tumor angiogenesis, and tumor progression. *J. Natl. Cancer Inst.* **89,** 219.
114. Bochner, B.H., Esrig, D., Groshen, S., et al. (1997) Relationship of tumor angiogenesis and nuclear p53 accumulation in invasive bladder cancer. *Clin. Cancer Res.* **3,** 1615.
115. Jaeger, T.M., Weidner, N., Chew, K., et al. (1995) Tumor angiogenesis correlates with lymph node metastases in invasive bladder cancer. *J. Urol.* **154,** 69.
116. Youusef, R.F., Shariat, S.F., Alhalabi, F., et al. (2010) Prospective validation of multiple biomarkers for improved clinical decision-making in patients with urothelial carcinoma of the bladder. AUA Meeting 2010 Abstracts. *J. Urol. Suppl.* **183,** e397.
